# The Close of the Volume

**Published:** 1892-12

**Authors:** 


### THE CLOSE OF THE VOLUME.

With the present number, Volume XIII. of the International Dental Journal will have completed the record for the year. Whether this marks an advance over those that have preceded it our readers are fully able to determine. It has been a year of continuous work, but it has had its compensation in that the effort has been in the direction of a higher standard of dental thought and practice. How far this may have reached, or to how many minds it may have been an incentive to better things, who may tell? It is not the prerogative of any editor to analyze his work. He can have little to do with results, but the motive that actuated thought is his own, and the mental compass, properly directed, will point unerringly in a true direction, and, this followed, will end in a safe harbor.

Exactly what constitutes the best-conducted journal has not yet been decided, and probably never can be to the satisfaction of all minds. The tastes of individuals are too various to ever harmonize. The editor, therefore, who aims to please all will end just where he ought to, in satisfying none. In all probability many have been displeased with certain portions of the work. We should be dissatisfied were it otherwise, as it would indicate that the duty intrusted to us had not been properly performed.

Progress can only be assured in any line of labor through friction. The man who never makes an enemy never can rise above the dead level of insipid thought, and the editor who proposes to live in harmony with all mankind is unfitted for his calling.

Without any appearance of egotism, we think the International Dental Journal has, month by month, furnished a most satisfactory exhibit of the higher mental and scientific work of the profession. We can point to this as proving that there has been a continued and healthy advance in intellectual force. Progress is made so slowly that it frequently appears as though no advance was being made, and it is only by comparison with past periods that just conclusions can be attained.

To those who have assisted the Journal by their valuable contributions we are deeply indebted. Without their aid it would have been forced to cease the work devolving upon it to perform. To those who have failed to recognize the importance of independent journalism we have this to say,—no great work has ever been performed in the world by men who lacked positive convictions, or who preferred expediency to principle.

The progress of the Journal the past year has been entirely satisfactory to the management. That this has been accomplished in the face of indifference and, at times, open antagonism, is certainly gratifying. As the only journal now published by dentists, and free from cliques and entangling alliances, it occupies the truest position to advance the best interests of those it aims to represent.

The changes the past year have not been many; indeed, so little has the surface of the professional waters been disturbed, that the ordinary observer would be inclined to believe that nothing had been gained, but this would be far from the truth.

The work, good or bad, is in the past. We have no promises to make for the future. The main desire will be to meet all demands, make the JOURNAL worthy of its readers, and, as new occasions arise, learn to advance with them to a still higher conception of the possibilities of professional life and professional principles.

